# Two cases of paraneoplastic limbic encephalitis associated with small cell lung cancer and a literature review

**DOI:** 10.3892/etm.2014.2142

**Published:** 2014-12-16

**Authors:** LI XU, JIANGUO HU, QIMING CHEN

**Affiliations:** 1 Department of Neurology, The First Affiliated Hospital of Bengbu Medical College, Bengbu, Anhui 233004, P.R. China; 2 Scientific Research Center, Bengbu Medical College, Bengbu, Anhui 233020, P.R. China

**Keywords:** paraneoplastic limbic encephalitis, anti-Hu antibodies, immunohistochemistry, western blotting

## Abstract

In the present study, two cases of paraneoplastic limbic encephalitis (PLE) associated with small cell lung cancer were reported. Using avidin-biotin immunoperoxidase methods, purified recombinant HuD western blotting and Euroline Neuronal Antigens Profile 2 immunoglobulin G western blotting, it was found that the well-characterized anti-Hu and anti-amphiphysin onconeuronal antibodies were present in the serum/cerebrospinal fluid of the patients. With a review of the literature, it was found that patients with PLE of Chinese Han nationality had two types of clinical manifestations, simple and complex, and that the lesions could also be divided into focal and scalable lesions. Furthermore, the clinical manifestations and lesion scopes were associated with certain types of cancer and antibodies. In addition, it was found that the prognosis for patients with PLE with autoantibodies targeting membrane antigens is improved compared with that for patients with PLE with autoantibodies targeting intracellular antigens, due to an increased sensitivity to immunomodulatory treatments and anti-cancer therapy.

## Introduction

Limbic encephalitis is a rare neurological syndrome that selectively affects the structures of the limbic system, including the hippocampus, amygdala and hypothalamus. Since the initial cases of this disease were often accompanied by cancer, such as small cell lung cancer (SCLC), the disease was subsequently referred to as paraneoplastic limbic encephalitis (PLE) (1–6). The main clinical manifestations of PLE are seizures associated with progressive short-term memory loss, which may develop into dementia. In addition, there may be different degrees of involvement in such extra-limbic-system tissues as the cerebellum, brainstem and thalamus. Electroencephalography (EEG) typically exhibits epileptic activity in the unilateral or bilateral temporal lobes, with focal or global slow waves; magnetic resonance imaging (MRI) T_2_ or flair images show high-signal, abnormal lesions in the interior sides of the unilateral or bilateral temporal lobes. In the majority of cases, temporal lobe atrophy develops. Cerebrospinal fluid examination typically exhibits inflammatory changes, with mildly to moderately increased lymphocytes, as well as increased protein levels; glucose levels would be normal and the immunoglobulin G (IgG) index would most likely be increased. Furthermore, oligoclonal bands would be apparent (7–9).

A number of studies have revealed that the pathogenesis of PLE is an immune-mediated response, primarily effected by cytotoxic T cells and antibodies that act on neuronal antigens, such as anti-Hu (10,11), anti-Ma2 (12), anti-amphiphysin (13) and anti-Yo (14) antibodies. Treatments for PLE include anti-cancer therapy and immunotherapy; the effects of the former are more marked, but the overall prognosis is typically poor (8,15). Research into PLE has made progress over the past decade (16), and certain clinical manifestations and imaging appearances have been found to be consistent with PLE. Furthermore, since the generation of antibodies targets neuronal cell membrane antigens, the development of specific immunotherapies could lead to improvements in prognosis for patients with PLE (15,17). To date, few studies have focused on PLE in patients of Chinese Han nationality; therefore, the present study described two cases of PLE in patients of Chinese Han nationality and summarized four cases in the literature.

## Materials and methods

### Clinical data

Two male patients with PLE associated with SCLC were hospitalized in the Department of Neurology, the First Affiliated Hospital of Bengbu Medical College (Bengbu, China) between October 1999 and July 2013. The patients were aged 69 and 83 years, respectively, and exhibited serious memory impairment, which prevented the patients from recalling the five designated objects presented on admission 5 min later. One of the patients complained of headache. One patient suffered from generalized tonic-clonic seizures (GTCSs), while the other suffered from complex partial seizures. The serum sodium levels of the two patients were as low as 115 and 130 mmol/l, and one patient had intractable hyponatremia. Cranial MRI was performed prior to treatment in both cases. This study was conducted in accordance with the Declaration of Helsinki and with approval from the Ethics Committee of Bengbu Medical College. Written informed consent was obtained from all participants.

### Wechsler Adult Intelligence Scale (WAIS) determination

A psychiatric doctor performed the test on the two patients prior to treatment (18). The language assessment included six aspects (knowledge test, comprehension, arithmetic, similarities, digit span and vocabulary) and the operation test consisted of five parts (number signs, picture filling, block design, picture arrangement and object assembly). The scores were obtained from the coarse score scale, and added to obtain the language IQ score, the operation IQ score and the total IQ points.

### Immunohistochemistry

The cerebral cortex and cerebellum were obtained from a neurologically normal individual within 6 h after mortality (the brain tissue was provided by Professor Zhou Jiangning from the School of Life Sciences, University of Sciences and Technology of China, Hefei, China). Consent was provided by the family of the deceased. Pieces were embedded in optimal cutting temperature compound and snap-frozen in isopentane cooled by liquid nitrogen, prior to being stored at −80°C. Tissue sections measuring 6 μm were sequentially incubated with 0.3% hydrogen peroxide (to block endogenous peroxidase activity) for 10 min and 10% normal goat serum (Organon Teknika-Cappel, West Chester, PA, USA) was then added as the blocking serum, prior to incubation for 15 min. The sera of the patients were serially diluted overnight at 4°C and then incubated with biotinylated goat anti-human IgG (Vector Laboratories, Burlingame, CA, USA) for 1 h and the Vectastain^®^ avidin-biotin complex (Vector Laboratories) for 30 min at room temperature. The substrate staining was developed with 0.05% diaminobenzidine tetraahydrochloride (Sigma, St Louis, MO, USA) and 0.01% hydrogen peroxide in phosphate-buffered saline (PBS).

### Western blotting

Western blotting was performed using the Euroline Neuronal Antigens Profile 2 IgG kit (DL1111-1601-2 G; Euroimmun AG, Lübeck, Germany). The film strip was removed and placed in the incubation tank. Sample buffer (1.5 ml) was added and incubated in a shaker (Euroimmun AG, Lübeck, Germany) at room temperature for 5 min, prior to the absorption of the liquid into the tank. Diluted serum sample (1.5 ml) was then added into the incubation vessel and agitated at room temperature (18–25°C) for 30 min incubation. Following incubation, the liquid was absorbed into the tank and the film strip was washed three times in a shaker with 1.5 ml washing buffer for 5 min/time. A total of 1.5 ml diluted enzyme conjugate (alkaline phosphatase-labeled anti-human IgG) was added into the incubation tank and then agitated in the shaker at room temperature for 30 min. The liquid was subsequently absorbed into the tank, and the film strip was washed in a shaker a further three times with 1.5 ml washing buffer for 5 min/time. Following washing, 1.5 ml substrate solution was added for another incubation at room temperature (18–25°C) for 10 min, prior to the liquid being absorbed into the tank and the film strip being washed three times with distilled water for 1 min/time. The film strip was then rotated in the result detecting template (Neuronal Antigens Profile 2; Euroimmun AG) for the analysis of the results following air-drying.

### Purified recombinant HuD

Purified recombinant HuD fusion protein, provided by Professor J.B. Posner (Department of Neurology, Memorial Sloan-Kettering Cancer Center, New York, NY, USA), was determined using the Bio-Rad Protein Assay (Bio-Rad Laboratories, Inc., Richmond, CA, USA). Samples were boiled in 0.0625 M Tris-HCl (pH 6.8), 2% sodium dodecyl sufate, 0.001% bromophenol blue and 5% 2-mercaptoethanol for 10 min. Aliquots (1 mg) were subsequently electrophoresed on preparative 10% sodium dodecyl sulfate-polyacrylamide gel, and the proteins were transferred to nitrocellulose membranes using the methodology described by Towbin *et al* (19). The membranes were then blocked with 5% Blotto (Carnation Company, Glendale, CA, USA) in PBS. The nitrocellulose membranes were cut into strips and incubated with the indicated amount of serum, which was diluted in buffer containing 1% bovine serum albumin, 10 mM Tris-HCl (pH 7.4), 0.9% sodium chloride and 0.5% Triton X-100 overnight at room temperature. Following incubation, the samples were washed four times for 15 min in the aforementioned buffer and incubated with biotinylated goat anti-human IgG (Vector Laboratories) for 1 h and the Vectastain avidin-biotin complex (Vector Labs) for 30 min at room temperature. The substrate staining was developed with 0.05% diaminobenzidine tetrahydrochloride (Sigma), 0.5% Triton X-100 and 0.01% hydrogen peroxide in PBS.

## Results

### WAIS determination

Prior to the treatment, the language IQ scores of the two patients were 45 and 57 points, the operation IQ scores were 42 and 43 points and the total IQ scores were 40 and 48 points. The patients scored poorly in the arithmetic, picture filling, block design, picture arrangement and object assembly.

### EEG

EEG revealed abnormalities in both cases, including focal or sharp slow waves in the bilateral frontotemporal lobes (Table I).

### Cranial MRI

**Figure 1:**
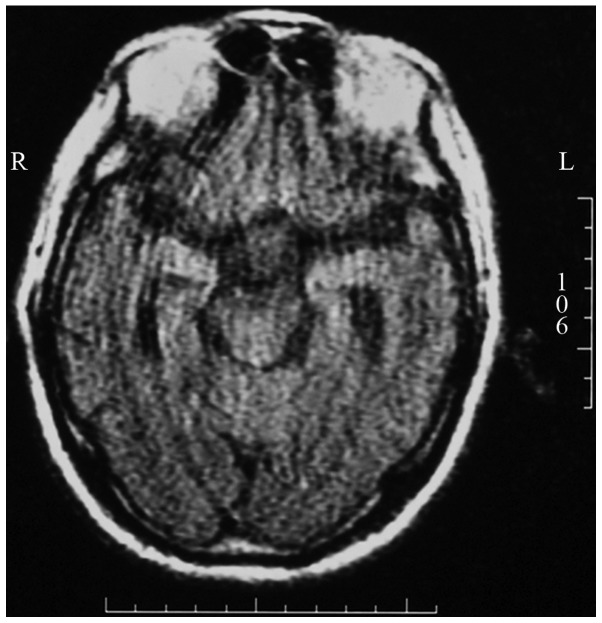
Head magnetic resonance imaging: One case had high signal intensity on the flair and T_2_-weighted image in the bilateral amygdala and hippocampal area.

One case had atrophy in the bilateral temporal lobe and hippocampal area and one case had high signal intensity on the flair and T_2_-weighted images in the bilateral amygdala and hippocampal area (Fig. 1).

### Immunohistochemistry

**Figure 2:**
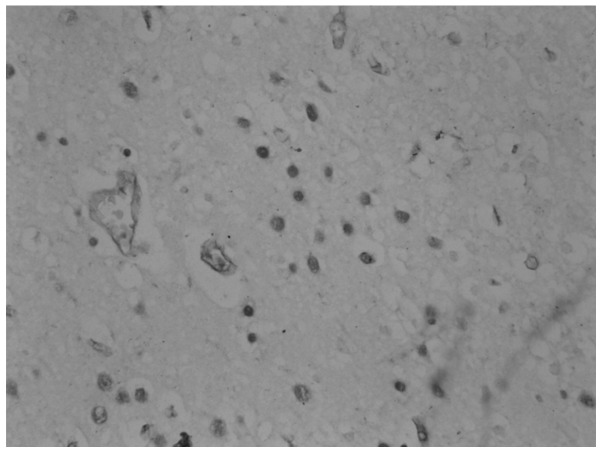
Section of frontal cortex incubated with a patient’s serum (dilution, 1:1,000). There is positive staining of the neuronal nuclei with a homogeneous pattern. The nucleoli are negative for staining. Original magnification, ×400.

The 69-year-old patient with PLE and SCLC was found to have anti-Hu antibodies in the serum. Following incubation of a section of the frontal cortex for 60 min with the patient’s serum [serum dilution, 1:1,000–1:16,000; cerebrospinal fluid (CSF) dilution, 1:100–1:800], positive staining of the neuronal nuclei was observed in a homogeneous pattern. No staining of the nucleoli was observed, and negative results were obtained for anti-Yo and -Ri antibodies (Fig. 2). The 83-year-old patient was found to have anti-amphiphysin antibodies in the serum. Anti-amphiphysin antibodies belong to the antibodies targeting the synaptic vesicle. Immunohistochemical tests are unable to show positive staining even in the presence of anti-amphiphysin antibodies.

### Western blotting

**Figure 3:**
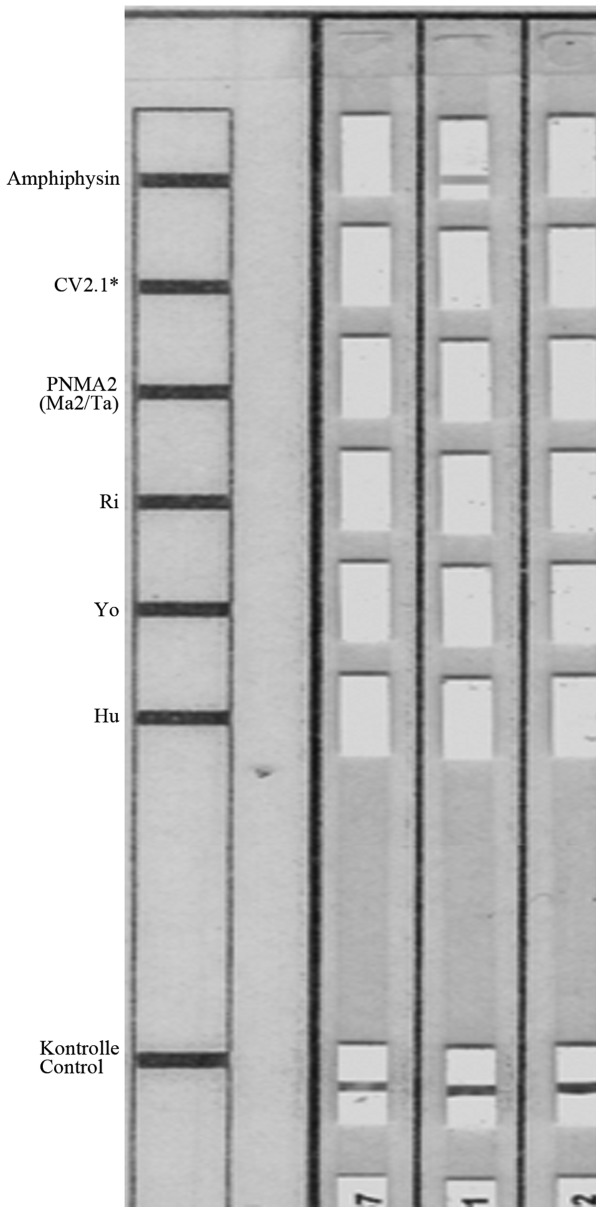
The paraneoplastic neuronal antibodiy spectrum examination included six specifically certified paraneoplastic neuronal antibodies, namely anti-Hu, anti-Yo, anti-Ri, anti-CV2, anti-PNMA2 (Ma2/Ta) and anti-amphiphysin. One patient with paraneoplastic limbic encephalitis and small cell lung cancer was positive for anti-amphiphysin antibody. PNMA2, paraneoplastic antigen Ma2.

The paraneoplastic neuronal antibody spectrum examination included six well-characterized onconeuronal antibodies: Anti-Hu, anti-Yo, anti-Ri, anti-CV2, anti-paraneoplastic antigen Ma2 (PNMA2) and anti-amphiphysin. One of the patients with PLE and SCLC was positive for anti-amphiphysin antibodies (Fig. 3).

### Purified recombinant HuD

**Figure 4:**
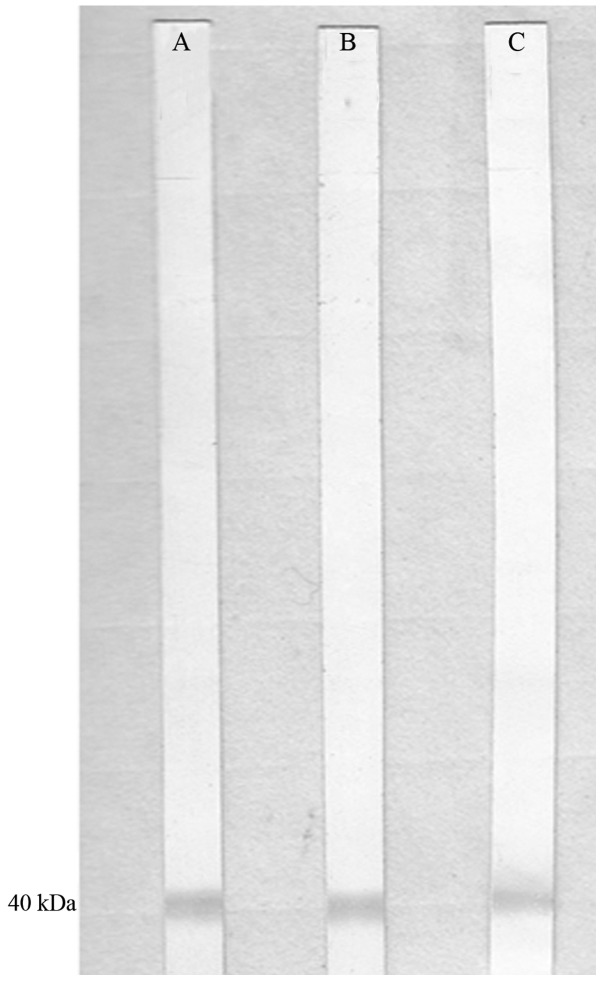
Immunoblots of purified recombinant HuD reacted with the serum of a patient with small cell lung cancer and paraneoplastic limbic encephalitis. Each lane is a different sample of the same patient’s serum; the dilutions are (A) 1:50, (B) 1:100 and (C) 1:200. The serum of the patient was shown to be positive for anti-Hu antibody (dilution, 1:50–1:200).

Immunoblots of purified recombinant HuD reacted with the serum of one of the patients with PLE and SCLC. The serum of the patient was positive for anti-Hu antibody (dilution, 1:50–1:200) (Fig. 4).

## Discussion

PLE is considered a rare manifestation that is characterized by the development of the neuropsychiatric symptoms of a paraneoplastic neurological disorder, but additionally associated with cancer in the absence of invasion of the nervous system by tumor cells. The tumor most commonly found in association with PLE is SCLC. The disease can affect individuals aged 10–85 years, with the females being less susceptible than males (5,20). Rarer malignancies associated with PLE are thymoma (21), ovarian teratoma (22,23), esophagastric squamous cell carcinoma (24), adenocarcinoma of the colon (14), prostate cancer (25), testicular neoplasm (26,27), Hodgkin’s lymphoma (20), non-Hodgkin’s lymphoma (28), leukemia, lymphoma (29) and acute myeloid leukemia (17). A literature search identified four studies containing data on cancer-related PLE in patients of Chinese Han nationality (30–33). Through the clinical manifestations, psychology, WAIS determination, CSF analysis, electrophysiology, imaging, immunological determination and anti-immunotherapy in the patients with PLE, it was found that the patients had two types of clinical manifestations, simple and complex, and that the lesions could also be divided into focal and scalable types. Of the four patients identified in the literature and the present two cases, three patients had PLE with SCLC (32), one case had PLE with pancreatic cancer (31) and two patients had PLE with ovarian teratoma (30,33). Four of the cases presented as an isolated neurological syndrome (progressive short-term memory loss, GTCSs) (32,33). One of the patients MRI showed the involvement of the bilateral frontal, right temporal and occipital lobes, as well as the left cerebellar hemisphere (31). PET-CT confirmed that the metabolism in the bilateral frontal, temporal, right parietal and occipital lobe was reduced. The patient had sucking, groping and grasping reflexes and a diffuse, brisk, deep tendon reflex (31,32). One case with PLE and SCLC had Lambert-Eaton myasthenic syndrome, and the lesion involved voltage-sensitive calcium channels of the presynaptic membrane in neuromuscular transmission (Table I).

The clinical diagnosis of PLE is problematic; however, the identification of a number of specific circulating autoantibodies, such as anti-Hu (34,1), anti-PNMA2 (12), anti-Yo (14), anti-N-methyl-D-aspartate receptor (NMDAR) (35) and anti-voltage-gated potassium channel (17) antibodies, in these patients has revolutionized the diagnosis and understanding of these syndromes and demonstrated a role for the immune system in such neurological disorders. We have speculated that the clinical manifestations and lesions scopes are associated with certain types of tumors and antibodies. In cases of autoantibodies targeting intracellular antigens (anti-Hu, anti-PNMA2, anti-Yo and anti-amphiphysin), an associated malignancy can nearly always be observed. These neurological disorders are predominantly associated with neuronal death, and patients are rarely sensitive to immunomodulatory treatments; cellular immunity appears to play a major role in this lack of sensitivity. By contrast, patients with autoantibodies targeting membrane antigens (receptors, channels or receptors associated with proteins) almost always have ovarian teratoma (21,35,33,30), and the neurological disorders are associated with a reversible neuronal dysfunction. These patients are mostly sensitive to immunomodulatory treatments, and it appears that humoral immunity and autoantibodies play a major role.

Using avidin-biotin immunoperoxidase methods, it was found in the present study that the serum of one patient with PLE and SCLC reacted with a section of frontal cortex (positive staining of the neuronal nuclei in a homogeneous pattern, but negative in the nucleoli). In purified recombinant HuD western blotting, the serum of one patient reacted with a band of 40-kDa purified recombinant HuD, a human neuronal RNA-binding protein (36), while Euroline Neuronal Antigens Profile 2 IgG western blotting showed the serum of the other patient to be positive for anti-amphiphysin antibodies. Anti-Hu and anti-amphiphysin are considered to be well-characterized onconeuronal antibodies, and their presence leads to the diagnosis of the PLE as classical paraneoplastic syndrome. Based on these considerations, the two patients in the present study developed definite paraneoplastic neurological syndrome (PNS) (37). Anti-amphiphysin antibodies have been identified in a few patients with PNS and SCLC, whose manifestations included encephalomyelitis/sensory neuropathy, cerebellar degeneration and opsoclonus (13). In the present study, the serum of one patient with PLE was positive for anti-amphiphysin antibody, which was rare. Despite the lack of evidence that these antibodies are the causative agents of the neuropathological process, they have proven to be useful markers of the autoimmune inflammation of structures in the limbic system. Dalmau *et al* (22) first described a number of cases of encephalitis with ovarian teratoma that were associated with the NMDAR antibodies, and subsequently analyzed the characteristics of 100 patients (35): 56% had ovarian teratoma, and the patients that received early tumor treatment and immunotherapy had an improved outcome. In a study by Xu *et al*, two cases had ovarian teratoma and one case had serum NMDAR antibodies (30). Tumor resection and immunotherapy resulted in a full recovery.

Patients with PLE of Chinese Han nationality had two types of clinical manifestation: simple and complex. Furthermore, the lesions could also be divided into focal and scalable lesions. The clinical manifestations and lesion scopes were associated with certain types of cancer and antibodies. Compared with patients with PLE with autoantibodies targeting intracellular antigens, the prognosis for patients with PLE with autoantibodies targeting membrane antigens is improved as a result of immunomodulatory treatments and anti-cancer therapy.’

## Figures and Tables

**Table I:** Clinical features of paraneoplastic limbic encephalitis.

Patient no. (ref.)	Gender, age in years	Tumor type/location	Symptoms and signs	EEG	CSF protein (mg/day)	Brain MRI and ^18^F-FDG/PET-CT	Neuronal antibodies	Prognosis
1 (present study)	M, 69	SCLC	Disorientation and GTCS for 90 days, Na^+^ 115 mmol/l	Bilateral frontal slow wave, right temporal lobe focal sharp-wave	Normal	Brain MRI: Atrophy in the bilateral temporal lobe and hippocampal area	Anti-Hu^+^	Mortality
2 (present study)	M, 83	SCLC	Progressive short-term memory loss, partial complex seizure Na^+^ 130 mmol/l	Bilateral frontal, right temporal lobe slow wave	Normal	MRI: High signal intensity on the flair and T_2_-weighted image in the bilateral amygdala and hippocampal area	Anti-amphiphysin^+^	Mortality
3 (31)	M, 49	Pancreatic cancer	Clumsy, apathy, echoing speech, memory loss, partial complex seizure; sucking, groping and grasping reflexes and a diffuse, brisk, deep tendon reflex; disorganization	Bilateral frontal, right temporal lobe, focal slow wave	Protein: 900 mg/l	MRI: Bilateral frontal lobe, temporal lobe, left parietal lobe, occipital lobe, cerebellar hemisphere, right parietal lobe. PET-CT: Bilateral frontal temporal lobe, right parietal lobe, occipital lobe, decreased metabolism	Negative	Mortality
4 (33)	F, 22	Ovarian teratoma	Apathy, babbing, conscious disturbance, GTCS status, Na^+^ 130 mmol/l	Bilateral diffuse slow wave	WBC: 16×10^6^/l Protein: 50 mg/l	Brain MRI: Normal	Negative	Recovery
5 (30)	F, 17	Ovarian teratoma	Short-term memory loss, emotional disturbance, GTCS status	Bilateral diffuse height amplitude δ waves	Intracranial hypertension, 220 mm H_2_O; WBC: 16×10^6^/l, Protein: 580 mg/l	Brain MRI: Normal	NMDAR^+^	Recovery
6 (32)	M, 52	SCLC	Short-term memory loss, partial complex seizure, Lambert-Eaton syndrome	-	-	MRI: Brain atrophy	Negative	Mortality

Ref., reference number; EEG, electroencephalography; CSF, cerebrospinal fluid; MRI, magnetic resonance imaging; ^18^F-FDG/PET-CT, ^18^F-fluorodeoxygluocose/positron emission tomography-computed tomography; SLCL, small cell lung cancer; GTCS, generalized tonic-clonic seizure; WBC, white blood cells; NMDAR, N-methyl-D-aspartate receptor.

## References

[1] Alamowitch S, Graus F, Uchuya M, Rene R, Bescansa E, Delattre JY (1997). Limbic encephalitis and small cell lung cancer Clinical and imunological features. Brain.

[2] Ryu JY, Lee SH, Lee EJ (2012). A case of paraneoplastic limbic encephalitis associated with small cell lung cancer. Tuberc Respir Dis (Seoul).

[3] Bowyer S, Webb S, Millward M, Jasas K, Blacker D, Nowa A (2011). Small cell lung cancer presenting with paraneoplastic limbic encephalitis. Asia Pac J Clin Oncol.

[4] Rosenfeld MR, Dalmau J (2007). Paraneoplastic limbic encephalitis associated with small-cell lung cancer. Comm Oncol.

[5] White D, Beringer T (2010). Paraneoplastic limbic encephalitis in an elderly patient with small cell lung carcinoma. Ulster Med J.

[6] Said S, Cooper CJ, Reyna E, Alkhateeb H, Diaz J, Nahleh Z (2013). Paraneoplastic limbic encephalitis, an uncommon presentation of a common cancer: Case report and discussion. Am J Case Rep.

[7] Urbach H, Soeder BM, Jeub M, Klockgether T, Meyer B, Bien CG (2006). Serial MRI of limbic encephalitis. Neuroradiology.

[8] Bien CG, Elger CE (2007). Limbic encephalitis: a cause of temporal lobe epilepsy with onset in adult life. Epilepsy Behav.

[9] Dreessen J, Jeanjean AP, Sindic CJ (2004). Paraneoplastic limbic encephalitis: Diagnostic relevance of CSF analysis and total body PET seanning. Acta Neurol Belg.

[10] Dalmau J, Furneaux HM (1990). Detection of the anti-Hu antibody in the serum of patients with small cell lung cancer - a quantitative western blot analysis. Ann Neurol.

[11] Langer JE, Lopes MB (2012). An unusual presentation of anti-Hu-associated paraneoplastic limbic encephalitis. Dev Med Child Neurol.

[12] Leyhe T, Schüle R, Schwarzler F, Gasser T, Haarmeier T (2006). Second primary tumor in anti-Mal/2-positive paraneoplastic limbic encephalitis. J Neurooncol.

[13] Saiz A, Dalmau J, Butler MH, Chen Q, Delattre JY, De Camilli P, Graus F (1999). Anti-amphiphysin I antibodies in patients with paraneoplastic neurological disorders associated with small cell lung carcinoma. J Neurol Neurosurg Psychiatry.

[14] Adam VN, Budincevic H, Mrsic V, Stojcic EG, Matolic M, Markic A (2013). Paraneoplastic limbic encephalitis in a patient with adenocarcinoma of the colon: a case report. J Clin Anesth.

[15] Dalmau J, Rosenfeld MR (2008). Paraneoplastic syndromes of the CNS. Lancet Neurol.

[16] Honnorat J, Viaccoz A (2011). New concepts in paraneoplastic neruological syndromes. Rev Neurol (Paris).

[17] Alcantara M, Bennani O, Verdure P, Leprêtre S, Tilly H, Jardin F (2013). Voltage-gated potassium channel antibody paraneoplastic limbic encephalitis associated with acute myeloid leukemia. Case Rep Oncol.

[18] Kaufman AS, Lichtenberger E (2006). Assessing Adolescent and Adult Intelligence.

[19] Towbin H, Staehelin T, Gordon J (1979). Electrophoretic transfer of proteins from polyacrylamide gels to nitrocellulose sheets: procedure and some applications. Proc Natl Acad Sci USA.

[20] Mollier-Saliner J, Thouvenin S, Darteyre S, Jaziri F, Vasselon C, Convers P, Stephan JL (2013). Paraneoplastic limbic encephalitis: 2 pediatric cases. Arch Pediatr.

[21] Ingenito GG, Berger JR, David NJ, Norenberg MD (1990). Limbic encephalitis associated with thymoma. Neurology.

[22] Dalmau J, Tüzün E, Wu HY (2007). Paraneoplastic anti-N-methyl-D-aspartate receptor encephalitis associated with ovarian teratoma. Ann Neurol.

[23] Kawano H, Hamaguchi E, Kawahito S, Tsutsumi YM, Tanaka K, Kitahata H, Oshita S (2011). Anaesthesia for a patient with paraneoplastic limbic encephalitis with ovarian teratoma: relationship to anti-N-methyl-D-aspartate receptor antibodies. Anaesthesia.

[24] McCormack O, Cooney JM, Doherty CP, Muldoon C, Reynolds JV (2013). Paraneoplastic limbic encephalitis from esophagogastric squamous cell carcinoma successfully managed by radical gastrectomy. Surgery.

[25] Jakobsen JK, Zakharia ER, Boysen AK, Andersen H, Schlesinger FE, Lund L (2013). Prostate cancer may trigger paraneoplastic limbic encephalitis: A case report and a review of the literature. Int J Urol.

[26] Ahern GL, O’Connor M, Dalmau J (1994). Paraneoplastic temporal lobe epilepsy with testicular neoplasm and atypical amnesia. Neurology.

[27] Burton GV, Bullard DE, Walther PJ, Burger PC (1988). Paraneoplastic limbic encephalopathy with testicular carcinoma a reversible neurologic syndrome. Cancer.

[28] Semnic M, Jovanovic D, Petrovic D, Nad I, Semnic R (2004). Paraneoplastic limbic encephalitis in a patient with non-Hodgkin’s lymphoma. Arch Oncol.

[29] Laffon M, Giordana C, Almairac F, Benchetrit M, Thomas P (2012). Anti-Hu-associated paraneoplastic limbic encephalitis in Hodgkin lymphoma. Leuk Lymphoma.

[30] Xu CL, Zhao WQ, Li JM (2010). Anti-N-methyl-D-aspartate receptor encephalitis: an adolescent with ovarian teratoma. Zhong Hua Shen Jing Ke Za Zhi.

[31] Zhang KZ, Wang Z, Chen WX, Song CJ (2008). Paraneoplastic limbic encephalitis in one patient with pancreatic cancer. Zhong Hua Shen Jing Ke Za Zhi.

[32] Zhao CP, Xie ZH, Peng CL, Wang Y, Sun L (2010). Paraneoplastic limbic encephalitis associated with Lamber-Eaton syndrome in one patient with small cell lung cancer. Zhong Guo Shen Jing Jing Shen Bing Xue Za Zhi.

[33] Zhou SN, Fu XZ, Liu YM (2009). Paraneoplastic limbic encephalitis in one patient with ovarian teratoma. Zhong Hua Shen Jing Ke Za Zhi.

[34] Graus F, Cordon-Cardo C, Posner JB (1985). Neuronal antinuclear antibody in sensory neuronopathy from lung cancer. Neurology.

[35] Dalmau J, Gleichman AJ, Hughes EG (2008). Anti-NMDA-receptor encephalitis: case series and analysis of the effects of antibodies. Lancet Neurol.

[36] Manley GT, Smitt PS, Dalmau J, Posner JB (1995). Hu antigens: reactivity with Hu antibodies, tumor expression, and major immunogenic sites. Ann Neurol.

[37] Graus F, Delattre JY, Antoine JC (2004). Recommended diagnostic criteria for paraneoplastic neurological syndromes. J Neurol Neurosurg Psychiatry.

